# Increased Rates of Hospitalized Children with Type 1 and Type 2 Diabetes Mellitus in Central Brooklyn during the COVID-19 Pandemic

**DOI:** 10.1155/2023/4580809

**Published:** 2023-04-17

**Authors:** Assia Miller, Shalu Joseph, Ahmed Badran, Vatcharapan Umpaichitra, Renee Bargman, Vivian L. Chin

**Affiliations:** ^1^Department of Pediatrics, Division of Pediatric Endocrinology, SUNY Downstate Health Sciences University, Brooklyn, NY 11203, USA; ^2^New York City/Health+Hospitals Corporation, Kings County Hospital, Brooklyn, NY 11203, USA

## Abstract

Following reports of increased new-onset diabetes and worse severity of DKA for children with diabetes following SARS-CoV-2 infection, we studied hospitalization rates for children with type 1 diabetes (T1DM) and type 2 diabetes (T2DM) in our center during the citywide shutdown. *Methods*. We conducted a retrospective chart review of children admitted to our two hospitals from January 1, 2018, to December 31, 2020. We included ICD-10 codes for diabetic ketoacidosis (DKA), hyperglycemic hyperosmolar syndrome (HHS), and hyperglycemia only. *Results*. We included 132 patients with 214 hospitalizations: 157 T1DM, 41 T2DM, and 16 others (14 steroid induced, 2 MODY). Overall admissions rates for patients with all types of diabetes were 3.08% in 2018 to 3.54% in 2019 (*p* = 0.0120) and 4.73% in 2020 (*p* = 0.0772). Although there was no increase of T1DM admissions across all 3 years, T2DM admission rates increased from 0.29% to 1.47% (*p* = 0.0056). Newly diagnosed T1DM rates increased from 0.34% in 2018 to 1.28% (*p* = 0.002) in 2020, and new-onset T2DM rates also increased from 0.14% in 2018 to 0.9% in 2020 (*p* = 0.0012). Rates of new-onset diabetes presenting with DKA increased from 0.24% in 2018 to 0.96% in 2020 (*p* = 0.0014). HHS increased from 0.1% in 2018 to 0.45% in 2020 (*p* = 0.044). The severity of DKA in newly diagnosed was unaffected (*p* = 0.1582). Only 3 patients tested positive for SARS-CoV-2 infection by PCR. *Conclusion*. Our urban medical center is located in Central Brooklyn and serves a majority who are Black. This is the first study investigating pediatric diabetes cases admitted to Brooklyn during the first wave of the pandemic. Despite the overall pediatric admissions declining in 2020 due to the citywide shutdown, overall hospitalization rates in children with T2DM and in new-onset T1DM and T2DM increased, which is not directly associated with active SARS-CoV-2 infection. More studies are needed to elucidate the reason for this observed increase in hospitalization rates.

## 1. Introduction

New York City (NYC) became an epicenter of the COVID-19 pandemic in March 2020, when the NYC Department of Health confirmed a total of 238,338 SARS-CoV-2 cases in NYC [1]. According to the NYC Department of Health, rates of SARS-CoV-2 cases and hospitalizations among children and adolescents ages 0-17 were 486.7 and 38.16 per 100,000 population during a pandemic, respectively [1]. Though these rates are low compared to older age categories, the CDC reported that children and adolescents with diabetes are at higher risk for severe COVID-19 disease [2].

The incidence of newly diagnosed diabetes continues to rise in the United States, with a 4.8% increase per year for type 2 diabetes (T2DM) and a 1.9% increase per year for type 1 diabetes mellitus (T1DM) between 2002 and 2015 [3]. The effect of SARS-CoV-2 infection may increase incidence of diabetes in children. Early on, as the first wave of the COVID-19 pandemic subsided, some have reported increased incidence of new-onset T1DM [3–9] and new-onset T2DM [6, 7, 10–12]. Initially, U.K. researchers noted an apparent rise of new-onset T1DM rates in children during the COVID-19 pandemic, with evidence of SARS-CoV-2 infection or exposure in a proportion of those tested [9]. Similarly, in the U.S., a recent single-center study discovered a 45% increase of new cases of T1DM [7]. Another U.S. study reported 15.2% rise of T1DM rates during the pandemic compared to prepandemic period, with non-Hispanic Blacks disproportionally affected over a three-year period [6].

Several researchers reported an increase of obesity risk factors and rates of obesity during lockdown and suggested that it may lead to metabolic decompensation, triggering the increase of T2DM [13–16]. Recent studies also found increased incidence and prevalence rates of presentation of T2DM [6, 11, 17–19]. One U.S. study found a significant rise of more than double the incidence (231%) of pediatric T2DM from 2019 to 2020 [7]. Interestingly, Marks et al. examined the variation by race and found that T2DM significantly increased during pandemic, with non-Hispanic Blacks (NHBs) disproportionally affected [6]. In contrast, Chao et al. found a steady rise in patients with new-onset T2DM, disproportionally affecting Hispanic youth (82%) [17].

In addition to the increasing cases, several authors reported the increase of risk and severity of diabetic ketoacidosis (DKA) for both T1DM and T2DM [3, 4, 6, 7, 9, 17, 20–23]. Though the exact cause is not known, it was suggested that delayed diagnosis of more than 30 days due to school closures may contribute to worsened severity of DKA [5, 24, 25]. In the U.K., authors emphasized that over half of children (52%) presented with severe DKA [9]. Recently, a meta-analysis examining the incidence of DKA in pediatric patients with T1DM concluded that compared to prepandemic, there was a 35% higher risk of DKA postpandemic (RR 1.35, 95% CI 1.2-1.35) and a 75% increased risk for severe DKA in T1DM (RR 1.76, 95% CI 1.33-2.33) [4]. Similarly, Marks et al. found not only increased rates of DKA in T1DM but also increased rates of severe DKA postpandemic [6]. In particular, NHB youth and boys had higher rates of incident T1DM during the pandemic than in the preceding two years [6]. A similar trend of increased DKA or DKA with hyperosmolality and severe DKA was observed in those with T2DM [6, 11, 17].

Racial disparities have contributed to poor outcomes in SARS-CoV-2 infection among NHB and Hispanic population; however, data on the incidence of new onset of T1DM and T2DM and variation by race, age, and severity of presentation in NHB youth in urban settings are not well studied. SUNY Downstate Health Sciences University and NYC H+H/Kings County are located in Central Brooklyn serving inner-city children and youth; therefore, data regarding diabetes admissions and severity of presentation during the COVID-19 pandemic is needed. In addition, we took into account how the “COVID-19-only” designation at SUNY Downstate Health Sciences University affected hospital admissions. We hypothesized that there is an increased rate of hospitalization of children and adolescents with T1DM and T2DM in 2020 during the COVID-19 pandemic compared to 2018 and 2019. The main objective of our study is to examine whether there is an increase in hospitalization rates and severity of presentation (DKA or HHS) and their risk factors of pediatric patients with T1DM and T2DM in our two hospital sites from 2018 to 2020 due to the COVID-19 pandemic.

## 2. Methods

### 2.1. Study Design

We conducted a retrospective chart review examining admission rates of patients with diabetes admitted for DKA, hyperglycemic hyperosmolar syndrome (HHS), or hyperglycemia to the SUNY Downstate Health Sciences University and NYC H+H Hospital/Kings County Hospital from January 1, 2018, to December 31, 2020, using ICD-10 diagnostic codes for T1DM, T2DM, HHS, mixed DKA/HHA, and hyperglycemia.

Our sites serve predominantly Black and Caribbean population, with about 20% living below poverty level [26]. Considering how COVID-19 pandemic affected our hospital admissions due to the NYC shutdown in 2020 and our academic institution SUNY Downstate being designed as COVID-19-only in 2020, we examined proportion of diabetic pediatric admissions to general pediatric admissions. Institutional Review Board (IRB) approval was obtained for this study (IRB protocol 1675462). They supervise all research in our institution and ensure that human research is ethical and valid.

### 2.2. Data Collection

We included children and adolescents ages 2-21 years old who were admitted to SUNY or Kings County for treatment of type 1 and type 2 diabetes mellitus for DKA, hyperglycemic hyperosmolar syndrome (HHS), or hyperglycemia from January 1, 2018, to December 31, 2020. We excluded those who did not meet inclusion criteria. Patients were all admitted for new-onset stabilization and diabetes education, presenting either with hyperglycemia or DKA or HHS, or are established patients with known T1DM or T2DM, and other types of DM requiring further management for hyperglycemic emergencies.

### 2.3. Variables

The diagnosis of T1DM was confirmed by presence of antibodies (GAD65, insulin antibodies, islet cell antibodies, and/or zinc transporter 8 antibodies) as markers of autoimmune *β*-cell destruction that usually leads to absolute insulin deficiency [27]. T2DM was diagnosed in patients without autoantibodies, with obesity and signs of insulin resistance [28].

We applied the diagnostic criteria for diabetes mellitus recommended by American Diabetes Association [28]. Patient were diagnosed if fasting plasma glucose (FPG) ≥ 126 mg/dL (7.0 mmol/L), or 2-hour plasma glucose ≥ 200 mg/dL (11.1 mmol/L) during oral glucose tolerance test or randomly in patients with classic symptoms of hyperglycemia [28].

We applied the International Society for Pediatric and Adolescent Diabetes (ISPAD) biochemical criteria for the diagnosis of diabetic ketoacidosis (DKA) comprised of hyperglycemia (blood glucose > 11 mmol/L (≈200 mg/dL)), venous pH < 7.3, and serum bicarbonate < 15 mmol/L, with ketonemia or ketonuria [29], and DKA severity as severe DKA (pH < 7.1, serum bicarbonate < 5 mmol/L), moderate DKA (pH 7.1-7.19, serum bicarbonate < 10 mmol/L), or mild DKA (pH 7.2-7.3, serum bicarbonate < 15 mmol/L) [29]. Diagnostic criteria for HHS comprised of plasma glucose concentration > 33.3 mmol/L (600 mg/dL), venous pH > 7.25 or arterial pH > 7.30, serum bicarbonate > 15 mmol/L, small ketonuria, absent to mild ketonemia, and effective serum osmolality > 320 mOsm/kg [29].

When serum osmolality was not measured, it was calculated based upon the calculation serum osmolality (mmol/kg): [2 × Na+] + [BUN/2.8] + [glucose/18]).

Variables used in these analyses included date of service, age in years, sex, race, ethnicity, anthropometrics, vital signs, and clinical characteristics including diagnosis of T1DM or T2DM or other type (MODY or steroid induced), complications such as DKA, HHS, hyperglycemia, new-onset or known diabetes, medications, length of PICU stay and inpatient floor stay, time to resolution of DKA or HHS, A1C, venous or arterial blood gases, urine and serum osmolality, chemistries, and evidence of current SARS-CoV-2 infection (PCR results). All patients were tested for SARS-CoV-2 infection in the emergency room regardless of symptoms, which was hospital policy.

### 2.4. Statistical Analysis

We applied descriptive statistics and summarized continuous variables using mean and standard deviation. We applied *t*-test with Bonferroni's adjustments for multiple testing. Categorical variables were analyzed using frequencies and percentages, and comparisons were made using a chi-squared test or Fisher's exact test. We set alpha to 0.05 for significance.

During SARS-CoV-2 peak in March 2020, pediatric admissions were greatly decreased, especially to SUNY Downstate. We accounted for this by calculating admission rates as a proportion of cases of patients with diabetes admitted divided by total pediatric admissions to the hospital in one-year period (2018, 2019, or 2020). Admission rates were calculated as number of annual cases divided in number of annual admissions per 100 admitted patients. Rate ratio (RR) was calculated as the ratio of rates of two groups R1/R2 was calculated with their Poisson's 95% confidence interval and associated *p* value [30]. We stratified results by type of diabetes. We calculated the admissions rates by year 2018 and 2019 and compared the rates over time compared with the year of the pandemic (2020). Statistical analysis was conducted using SAS Institute Inc. (2013), Version 9.4.

## 3. Results

We included 132 patients with 214 hospitalizations: 157 T1DM, 41 T2DM, and 16 other (14 steroid induced, 2 MODY). In the two hospitals, there were 2078 admissions in 2018, there were 2146 admissions in 2019, and there were 1564 admissions of patients aged 2-21 in 2020.

### 3.1. Admission Rates

Overall diabetic admission rates increased from 3.08% in 2018 to 3.54% in 2019 and 4.73% in 2020, which was only significant between 2019 and 2020 (*p* = 0.012) (Table 1). However, by excluding steroid induced and MODY admissions, increases for T1DM and T2DM combined were significant between 2018 to 2020 and 2019 to 2020 (Table 1).

Though there was no difference in total T1DM admissions across all 3 years, T2DM admission rates increased dramatically from 0.29% (CI% 0.1-0.6) in 2018 and 0.56% (95% CI 0.3-1.0) in 2019 to 1.47% (CI% 0.9-2.2) (*p* = 0.0056) in 2020. Despite lower total pediatric cases in 2020, many more patients (*n* = 23) were admitted in 2020 with T2DM compared with 2018 (*n* = 6) and 2019 (*n* = 12). In 2020, 14 admissions were due to new-onset T2DM and 5 were due to DKA and 7 had HHS/mixed diagnosis.

We observed that new-onset DM hospitalization rates in 2018 was 0.53% and in 2019 was 0.84% and increased significantly to 2.17% in 2020. In terms of cases, the number of patients with new-onset DM increased from 11 to 18 to 34 from 2018 to 2019 to 2020. This increase is due to observed increases in both T1DM and T2DM new-onset admissions.

Newly diagnosed T1DM rates significantly increased from 0.34% in 2018 to 1.28% in 2020 (*p* = 0.0013), and new-onset T2DM rates also increased from 0.14% in 2018 to 0.9% (*p* = 0.0012) in 2020.

New-onset diabetes presenting with DKA also was noted to increase from 0.24% in 2018 to 0.96% in 2020 (*p* = 0.0014). We had 24 patients with new-onset diabetes presented with DKA admitted over 3-year time period. Five (21%) with DKA in 2018, 4 (17%) with DKA in 2019, and 15 (62%) with DKA in 2020 were admitted, indicating a 3-fold increase of admissions in 2020. Within this new-onset group presenting in DKA, the majority was ultimately diagnosed with T1DM (*n* = 10) compared to T2DM (*n* = 5). HHS/mixed presentation increased from 0.1% to 0.14% to 0.45% in 2018, 2019, and 2020, respectively.

In 2020, we observed an increase in HHS/mixed DKA cases which were accounted for by new-onset diagnosis of DM (*n* = 5), in contrast to 1 admission in 2018-2019. In 2020, from 7 total admissions for HHS/mixed DKA, 4 admissions were for T2DM, compared to none in 2018-2019.

Only 3 patients tested positive for COVID-19 by PCR testing on admission. One case was admitted with HHS/mixed DKA and SARS-CoV-2 pneumonia, another was admitted with hyperglycemia due to T2DM and SARS-CoV-2 pneumonia, and the last case was admitted with severe DKA and found to be SARS-CoV-2 positive.

### 3.2. Demographic Characteristics of Type 1 Diabetes

From 2018 to 2020, patients with T1DM were majority females (68%), Black (87%), and non-Hispanic (87%). There were no differences in demographic characteristics between years (Table 2).

### 3.3. Clinical Characteristics of Patients with Type 1 Diabetes

In 2020, patients presented with more severe acidosis (lower serum bicarbonate) when compared to 2018 (Table 3). Patients in 2020 were younger than patients in 2019 (*p* = 0.01), however had lower respiratory rates and lower BUN, but similar BMI *z*-scores.

### 3.4. Demographic Characteristics of Patients with Type 2 Diabetes

In contrast, patients admitted as T2DM mostly comprised of males (59%), with a majority of Black (95%) and non-Hispanic (95%) (Table 4).

### 3.5. Clinical Characteristics of Patients with Type 2 Diabetes

In 2020, patients were older and heavier (*p* = 0.034) and had higher HR (*p* = 0.01), higher glucose levels (*p* ≤ 0.001), and more severe acidosis (serum bicarbonate) (*p* = 0.04) compared to the previous years (Table 5). The degree of dehydration is also worse in 2020 given the higher creatinine levels. There was no difference in average A1C. Severity of DKA in newly diagnosed diabetes was unaffected (*p* = 0.1582) (data not shown).

## 4. Discussion

Consistent with the previous reports in the U.S. and international studies, we also observed a dramatic increase in hospitalization rates for new-onset DM in both type 1 and type 2 groups [5, 9]. Previous reports described that the diabetes incidence among patients age < 18 years was significantly higher among those with SARS-CoV-2 than among those without SARS-CoV-2 [5, 9]; however, we did not find a direct association of increased rates of DM with SARS-CoV-2 infection since we only had 3 active cases of SARS-CoV-2 detected via PCR upon admission. One limitation to our study is the relatively small sample size (referrals to our hospital were also limited by COVID-19-only designation) and limited to our center which only involves two hospitals. Additionally, we did not systematically investigate IgG antibody levels (to indicate recent, not active infection) or determine whether patients had recent symptoms suggesting prior SARS-CoV-2 infection.

In our study, the hospitalization rates of new-onset DM with DKA increased from 0.24 (95% CI 0.1-0.6) and 0.19 (95% CI 0.1-0.5) to 0.96 (95% CI 0.5-1.6) (*p* = 0.0014), similar to the previous reports [5, 6, 9, 17, 23, 31]. Similar to reports from the U.K. and Germany, we observed an increase of DKA severity (by lower serum bicarbonate levels) in 2020 compared to 2018 [5, 9, 23], particularly in T1DM. In T2DM, the degree of dehydration and hyperglycemia appeared worse in 2020, probably due to the increased number of patients presenting with mixed HHS/DKA, thus leading to longer time in DKA and recovery time in the PICU.

A previous study showed that DKA severity is worse among Black patients compared to others; however in our study, we had a majority of Black patients and were not able to do comparisons by race [6].

We did not find a significant increase of T1DM admission rates across 3 years. Our results are similar with the report by Marks et al. but is not in agreement with Unsworth et al. who observed an increase of T1DM cases in the U.K. [9]. These differences may be explained by a different demographic makeup of our study [6, 9]. Similar to other reports, we observed an increase of hospitalizations of new-onset T1DM [4, 6, 7, 9] and increased rates of patients hospitalized with DKA and new-onset T1DM [4, 23]. An increase in hospitalization rates for T2DM overall and new-onset T2DM is similar with other studies [6, 7, 12, 17]. We are unable to conclude whether there was a change in severity of DKA for T2DM during the COVID-19 pandemic, given that the numbers are quite small. We similarly found increased number of hospitalized patients with HHS/mixed picture, which is similar to other reports of increased severe DKA with hyperosmolarity during the COVID-19 pandemic in the U.S. [11, 12, 17].

We did not specifically look at longitudinal changes in BMI in the same cohort, although the observed BMI *z*-scores based on our data was not different in our study from prepandemic to pandemic years. In another study examining the changes in BMI *z*-score in pediatric patients in municipal hospitals across NYC, there was an increase in annual BMI *z*-score from 2018 to 2019 by 3.1% and from 2019 to 2020 by 34.3% in the general pediatric population [13] which helps lend suspicion that perhaps the observed increased hospitalizations of T2DM or new-onset T2DM may be due to weight changes.

Major strengths of our study are worth mentioning. Our cases were reviewed, and diagnosis and classification were based on ADA and ISPAD criteria. We examined a wide range of sociodemographic, clinical, and biochemical characteristics. Our sample was drawn from two hospitals in serving Central Brooklyn with a unique population of mostly Blacks and Caribbean-Americans. Admission rates for patients with diabetes were adjusted for overall decreases in pediatric cases which were affected by citywide shutdown as well as SUNY Downstate redesignation as COVID-19-only, rather than counting number of cases only. However, it is worth mentioning that we were unable to take into account changes in who was not coming into the Downstate ER and where they are receiving care and whether they stayed home or presented to another hospital in Brooklyn. This limitation is a minor cause of concern since the overall number of admissions for patients with diabetes and its breakdown shown in Table 1 shows the absolute number of patients increasing in conjunction with the calculated rates of hospitalizations increasing.

Additional limitation to this study was that we were not able to examine precise weight gain before admission for hospitalized patients, especially for new-onset T2DM. Initially, we were expecting to find that rapid or recent significant weight gain during the pandemic would be a risk factor for developing T2DM. Our sample was relatively homogeneous, with no opportunity to examine variation of admission rates by race and ethnicity.

## 5. Conclusion

Our urban academic medical center located in Central Brooklyn serves a majority who are Black. As far as we know, this is the first study investigating pediatric diabetes cases admitted in Central Brooklyn during the first major wave of the pandemic. Overall, hospitalization rates in children with T2DM and in new-onset T1DM and T2DM increased, despite the overall pediatric admissions declining in 2020 during the citywide shutdown. Patients with new-onset T1DM also presented with DKA more often than what we had seen prepandemic. Whether the shutdown affected patient's perception of their symptoms or another reason leading to delayed care or change in access to care remains to be seen. Active SARS-CoV-2 infection did not appear to be a risk factor for those admitted for diabetes. Larger-scale multicenter studies are needed to elucidate the reason for this observed increase in hospitalization rates of pediatric patients with diabetes mellitus.

## Figures and Tables

**Table 1:** Hospitalization rates of children and adolescents with type 1 and type 2 diabetes mellitus in our center from 2018 to 2020 (prepandemic to pandemic).

	Year 2018		Year 2019		Compare 2018-2019	Year 2020		Compare 2018-2020	Compare 2019-2020
	*N*	Rate^∗^ (95% CI)	*N*	Rate (95% CI)	RR^∗∗^ (95% CI) *p* value	*N*	Rate (95% CI)	RR (95% CI) *p* value	RR (95% CI) *p* value
Type 1 DM (*N* = 157)	52	2.50 (1.9-3.3)	57	2.66 (2.0-3.4)	1.06 (0.72-1.58) *p* = 0.76	48	3.07 (2.3-4.1)	1.23 (0.81-1.85) *p* = 0.31	1.16 (0.77-1.73) *p* = 0.46
Type 2 DM (*N* = 41)	6	0.29 (0.1-0.6)	12	0.56 (0.3-1.0)	1.94 (0.67-6.23) *p* = 0.19	23	1.47 (0.9-2.2)	5.09 (2.02-15.29) *p* = 0.0001	2.63 (1.26-5.80) *p* = 0.0056
DKA (*N* = 129)	37	1.78 (1.3-2.5)	50	2.33 (1.7-3.1)	1.31 (0.84-2.10) *p* = 0.22	42	2.69 (1.9-3.6)	1.51 (0.95-2.41) *p* = 0.07	1.15 (0.75-1.80) *p* = 0.50
New-onset DM (*N* = 63)	11	0.53 (0.3-1.0)	18	0.84 (0.5-1.3)	1.58 (0.71-3.71) *p* = 0.23	34	2.17 (1.5-3.0)	4.11 (2.03-8.99) *p* < 0.0001	2.59 (1.42-4.87) *p* = 0.0008
New-onset T1DM (*N* = 32)	7	0.34 (0.1-0.7)	5	0.23 (0.1-0.54)	0.69 (0.17-2.53) *p* = 0.54	20	1.28 (0.8-2.0)	3.80 (1.54-10.63) *p* = 0.0013	5.49 (2.00 -18.71) *p* = 0.0002
DKA new-onset T1DM (*N* = 16)	4	0.19 (0.1-0.5)	2	0.09 (0.01-0.3)	0.48 (0.04-3.38) *p* = 0.43	10	0.64 (0.3-1.2)	3.3216 1.001-14.51 *p* = 0.04	6.8606 (1.46-64.40) *p* = 0.005
New-onset T2DM (*N* = 24)	3	0.14 (0.03-0.4)	11	0.51 (0.3-0.9)	3.55 (0.94-19.82) *p* = 0.04	14	0.90 (0.5-1.5)	6.20 1.73-33.65 *p* = 0.0012	1.75 (0.74-4.25) *p* = 0.17
DKA new-onset type 2 (*N* = 8)	1	0.05 (0.001-0.3)	2	0.09 (0.01-0.3)	1.94 (0.10-114) *p* = 0.64	5	0.32 (0.1-0.7)	6.64 (0.74-314) *p* = 0.06	3.43 (0.56-36.02) *p* = 0.14
New-onset DKA (*N* = 24)	5	0.24 (0.1-0.6)	4	0.19 (0.1-0.5)	0.77 (0.15-3.60) *p* = 0.72	15	0.96 (0.5-1.6)	3.99 (1.38-14.02) *p* = 0.0046	5.15 (1.64-21.30) *p* = 0.0014
HHS/mixed (*N* = 12)	2	0.10 (0.01-0.3)	3	0.14 (0.03-0.4)	1.45 (0.17-17.4) *p* = 0.71	7	0.45 (0.2-0.9)	4.65 (0.89-45.9) *p* = 0.0444	3.20 (0.73-19.19) *p* = 0.0902
Hyperglycemia (*N* = 73)	25	1.20 (0.8-1.8)	23	1.07 (0.7-1.6)	0.89 (0.48 -1.64) *p* = 0.69	25	1.60 (1.0-2.4)	1.33 (0.73-2.41) *p* = 0.32	1.49 (0.81-2.75) *p* = 0.17
All T1DM and T2DM	58	2.8 (2.1-3.6)	69	3.2 (2.5-4.1)	1.15 (0.80-1.66) *p* = 0.43	71	4.5 (3.5-5.7)	1.6264 (1.13-2.34) *p* = 0.0060	1.41 (1.01-2.0) *p* = 0.0420
All cases	64	3.08 (2.4-3.9)	76	3.54 (2.8-4.4)	1.15 (0.81-1.63) *p* = 0.41	74	4.73 (3.7-5.9)	1.54 (1.08-2.18) *p* = 0.0120	1.34 (0.96-1.86) *p* = 0.0772
Total pediatric admissions		2078		2146			1564		

Rate^∗^: admission rate. RR^∗∗^: rate ratio.

**Table 2:** Demographic characteristics of children and adolescents with type 1 diabetes mellitus admitted during COVID-19 pandemic.

	Year 2018		Year 2019		Year 2020		Total		*p* value
	*N*	%	*N*	%	*N*	%	*N*	%	
Total	52	33%	57	36%	48	31%	157	100%	
Sex									
Female	33	63%	37	65%	37	77%	107	68%	0.2771
Male	19	37%	20	35%	11	23%	50	32%	
Race									
Black	44	85%	49	86%	45	94%	138	87%	0.2849
White	7	13%	8	14%	2	12%	17	11%	
Asian	0	0%	0	0%	1	2%	1	1%	
Unknown	1	2%	0	0%	0	0%	1	1%	
Ethnicity									
Hispanic	7	4%	7	4%	3	2%	17	10%	0.4505
Non-Hispanic	43	83%	49	86%	45	94%	137	87%	
Unknown	2	1%	1	1%	0	0%	3	3%	

**Table 3:** Clinical characteristics of children and adolescents with type 1 diabetes mellitus admitted during COVID-19 pandemic.

	Year 2018	Year 2019	Compare 2018-2019	Year 2020	Compare 2018-2020	Compare 2019-2020
	Mean (SD)	Mean (SD)	*p* value	Mean (SD)	*p* value	*p* value
Age (years)	13 (3.8)	16 (3.8)	0.0001	13.8 (4.7)	0.34	0.01
Length of hospitalization (days)	3.7 (1.7)	3.4 (1.5)	0.32	3.6 (1.5)	0.67	0.54
Length of PICU stay (days)^∗^	1.5 (0.8)	1.6 (0.7)	0.40	1.4 (1.0)	0.74	0.27
Length of floor stay (days)^∗∗^	2.2 (1.3)	1.9 (1.5)	0.35	2.1 (1.4)	0.62	0.68
Duration of DKA (hours)^∗∗∗^	17.1 (10.0)	16.8 (10)	0.87	14.1 (7.2)	0.13	0.99
*Anthropometrics*						
Weight (kg)	50.0 (18.1)	57.1 (15.9)	0.03	49.8 (18.9)	0.95	0.03
Height (cm)	153.6 (18.9)	159.5 (26.2)	0.18	151.8 (20.6)	0.65	0.10
BMI (kg/m^2^)	20.4 (4.6)	21.1 (3.1)	0.40	23.3 (21.2)	0.34	0.43
BMI percentile (%)	55.6 (31.9)	56.9 (24.5)	0.81	49.8 (32.7)	0.38	0.22
BMI *z*-score	0.1 (1.2)	0.1 (0.9)	0.99	-0.1 (1.6)	0.50	0.44
*Vital signs*						
Heart rate (beat per min)	116.4 (19.7)	117.5 (18.2)	0.75	115.8 (18.9)	0.75	0.63
Respiratory rate (breath per min)	23.9 (7.)8)	23.0 (5.7)	0.47	20.8 (3.1)	0.47	0.02
Temperature (°F)	98.4 (0.8)	98.2 (0.7)	0.15	98.5 (1.0)	0.15	0.05
Oxygen (%)	99.2 (1.1)	97.8 (13.1)	0.45	99.2 (1.0)	0.45	0.48
*Biochemistry*						
Glucose (mg/dL)	488.2 (243)	501.3 (201)	0.76	545.4 (272)	0.76	0.34
Sodium (mmol/L)	135.0 (4.4)	132.9 (5.1)	0.02	132.3 (4.8)	0.02	0.54
Potassium (mmol/L)	5.3 (1.3)	5.4 (1.5)	0.69	5.2 (1.3)	0.69	0.53
Chloride (mmol/L)	96.4 (9.3)	95.3 (7.9)	0.49	95.9 (6.6)	0.49	0.68
Bicarbonate (CO_2_) (mmol/L)	13.4 (6.3)	10.1 (6.4)	0.02	12.7 (6.9)	0.02	0.08
BUN (mg/dL)	18.6 (9.2)	17.4 (6.8)	0.42	14.8 (5.6)	0.42	0.04
Creatinine (mg/dL)	1.0 (0.4)	1.1 (0.4)	0.16	1.0 (0.3)	0.16	0.15
Calcium (mg/dL)	9.6 (2.2)	9.7 (1.7)	0.72	9.0 (3.0)	0.72	0.12
Phosphorus (mg/dL)	5.1 (2.3)	4.8 (2.2)	0.65	4.8 (2.4)	0.65	0.95
Magnesium (mg/dL)	2.3 (0.6)	2.2 (0.4)	0.81	2.2 (0.7)	0.81	0.98
HbA1c (%)	12.1 (2.0)	12.6 (2.2)	0.24	13.1 (2.2)	0.24	0.38
Serum osmolality (mOsm/kg)	312 (40)	307 (20)	0.37	307 (27)	0.42	0.99
Beta-hydroxybutyrate (BHOB) (mmol/L)	5.3 (2.9)	6.5 (3.3)	0.08	5.6 (2.4)	0.08	0.13
Urine ketone	83.5 (40.7)	74.5 (17.4)	0.11	67.2 (31.8)	0.62	0.18
*Venous blood gas*						
pCO_2_ (mmHg)	34.5 (10. 6)	28.1 (10.8)	0.0027	32.4 (9.9)	0.00	0.04
Bicarbonate (HCO_3_) (mmol/L)	15.0 (6.9)	12.1 (5.8)	0.0214	14.8 (6.5)	0.02	0.03
pH	7.21 (0.1)	7.13 (0.2)	0.0029	7.18 (0.2)	0.35	0.09

Length of PICU stay (days)^∗^ was calculated only among cases who were managed in PICU. Length of floor stay (days)^∗∗^ was calculated only among cases who were managed on the floor. Duration of DKA (hours)^∗∗∗^ was calculated only among cases admitted with DKA.

**Table 4:** Demographic characteristics of children and adolescents with type 2 diabetes mellitus admitted during COVID-19 pandemic.

	Year 2018		Year 2019		Year 2020		Total		*p* value
	*N*	%	*N*	%	*N*	%	*N*	%	
	6	15%	12	29%	23	56%	41	100%	
Sex									
Female	2	33%	7	58%	8	35%	17	41%	0.3691
Male	4	67%	5	42%	15	65%	24	59%	
Race									
Black	5	83%	12	100%	22	96%	39	95%	0.2973
White	0		0		0		0		
Asian	1	17%	0	0	1	4%	2	5%	
Unknown	0		0		0		0		
Ethnicity									
Hispanic	0		0		0		0		
Non-Hispanic	6	100%	10	83%	23	100%	39	95%	0.0788
Unknown	0	0	2	17%	0	0%	2	5%	

**Table 5:** Clinical characteristics of children and adolescents with type 2 diabetes mellitus admitted during COVID-19 pandemic.

*N*	Year 2018	Year 2019	Compare 2018-2019	Year 2020	Compare 2018-2020	Compare 2019-2020
	Mean (SD)	Mean (SD)	*p* value	Mean (SD)	*p* value	*p* value
Age mean (years)	13.2 (2.7)	12.9 (3.4)	0.87	15.8 (3.1)	0.07	0.02
Length of hospitalization (days)	3.2 (0.8)	3.2 (1.1)	0.86	3.7 (2.0)	0.56	0.35
Length of PICU stay (days)^∗^	1.5 (0.7)	1.0 (1.0)	0.67	1.79 (1.3)	0.78	0.59
Length of floor stay (days)^∗∗^	2.3 (1.5)	3.0 (1.1)	0.38	2.3 (1.5)	0.93	0.16
Duration of DKA (hours)^∗∗∗^	13.5 (7.8)	9.0 (0.0)	0.33	22.0 (16.2)	0.46	0.44
*Anthropometrics*						
Weight (kg)	85.0 (43.2)	86.1 (35.9)	0.03	98.7 (31.8)	0.95	0.03
Height (cm)	160.5 (15.9)	164.2 16.4)	0.18	161.2 (37.0)	0.65	0.09
BMI (kg/m^2^)	31.6 (11.1)	30.8 (7.5)	0.86	34.6 (8.4)	0.48	0.21
BMI percentile (%)	94.2 (8.6)	95.4 (7.1)	0.74	97.5 (3.3)	0.15	0.26
BMI *z*-score	2.0 (0.8)	2.1 (0.7)	0.88	2.2 (0.6)	0.40	0.38
*Vital signs*						
Heart rate (beat per min)	86.7 (13.0)	88.1 (13.1)	0.83	109.1 (22.4)	0.83	0.01
Respiratory rate (breath per min)	18.7 (2.4)	19.3 (1.1)	0.49	20.1 (3.1)	0.49	0.38
Temperature (°F)	98.5 (0.3)	98.1 (1.3)	0.41	98.5 (1.1)	0.41	0.34
Oxygen %	99.5 (0.8)	99.5 (1.2)	1.00	98.3 (2.4)	1.00	0.13
*Biochemistry*						
Glucose (mg/dL)	377 (178)	313 (75.6)	0.29	546.5 (252.2)	0.14	0.00
Sodium (mmol/L)	136.5 (2)	134.1 (3.4)	0.14	134.5 (4.3)	0.29	0.76
Potassium (mmol/L)	4.2 (0.2)	4.2 (0.3)	0.53	4.5 (0.8)	0.30	0.33
Chloride (mmol/L)	99.7 (2.2)	97.8 (4.0)	0.32	97.1 (6.7)	0.37	0.75
Bicarbonate (CO_2_) (mmol/L)	27.2 (0.2)	22.4 (2.6)	0.054	16.0 (8.1)	0.01	0.04
BUN (mg/dL)	9.2 (1.5)	11.9 (3.4)	0.08	15.1 (7.8)	0.08	0.21
Creatinine (mg/dL)	0.9 (0.4)	0.8 (0.2)	0.51	1.2 (0.7)	0.20	0.02
Calcium (mg/dL)	9.5 (0.6)	9.9 (0.5)	0.11	9.8 (2.2)	0.68	0.91
Phosphorus (mg/dL)	3.5 (1.2)	4.6 (0.7)	0.10	4.6 (1.6)	0.23	0.92
Magnesium (mg/dL)	1.9 (0.2)	2.0 (0.2)	0.81	2.2 (0.6)	0.32	0.38
HbA1c (%)	13.0 (2.0)	11.6 (2.6)	0.28	12.3 (2.1)	0.45	0.45
Serum osmolality (mOsm/kg)	305.1 (16)	292.6 (9.3)	0.046	318.0 (46)	0.51	0.08
Beta-hydroxybutyrate (BHOB) (mmol/L)	1.1 (0.7)	2.4 (2.5)	0.40	4.1 (3.5)	0.16	0.26
Urine ketone	60.0 (28)	40.0 (38.7)	1.00	55.7 (31.4)	1.00	1.00
*Venous blood gas*						
pCO_2_ (mmHg)	44.6 (10.9)	42.1 (6.9)	0.59	37.3 (10.0)	0.16	0.18
Bicarbonate (HCO_3_) (mmol/L)	23.5 (8.1)	22.4 (3.6)	0.71	17.4 (7.8)	0.10	0.06
pH	7.26 (0.2)	7.35 (0.05)	0.08	7.23 (0.2)	0.78	0.04

Length of PICU stay (days)^∗^ was calculated only among cases who were managed in PICU. Length of floor stay (days)^∗∗^ was calculated only among cases who were managed on the floor. Duration of DKA (hours)^∗∗∗^ was calculated only among cases admitted with DKA.

## Data Availability

The original data used to support the findings of this study is available from the corresponding author upon request.
